# Metamorphosis revealed: time-lapse three-dimensional imaging inside a living chrysalis

**DOI:** 10.1098/rsif.2013.0304

**Published:** 2013-07-06

**Authors:** Tristan Lowe, Russell J. Garwood, Thomas J. Simonsen, Robert S. Bradley, Philip J. Withers

**Affiliations:** 1 The Manchester X-Ray Imaging Facility, School of Materials, University of Manchester, Manchester M13 9PL, UK; 2 School of Earth, Atmospheric and Environmental Sciences, University of Manchester, Manchester M13 9PL, UK; 3 Department of Life Sciences, Natural History Museum, Cromwell Road, London SW7 5BD, UK

**Keywords:** insect development, micro-CT, Lepidoptera, metamorphosis, time-lapse, *Vanessa cardui*

## Abstract

Studies of model insects have greatly increased our understanding of animal development. Yet, they are limited in scope to this small pool of model species: a small number of representatives for a hyperdiverse group with highly varied developmental processes. One factor behind this narrow scope is the challenging nature of traditional methods of study, such as histology and dissection, which can preclude quantitative analysis and do not allow the development of a single individual to be followed. Here, we use high-resolution X-ray computed tomography (CT) to overcome these issues, and three-dimensionally image numerous lepidopteran pupae throughout their development. The resulting models are presented in the electronic supplementary material, as are figures and videos, documenting a single individual throughout development. They provide new insight and details of lepidopteran metamorphosis, and allow the measurement of tracheal and gut volume. Furthermore, this study demonstrates early and rapid development of the tracheae, which become visible in scans just 12 h after pupation. This suggests that there is less remodelling of the tracheal system than previously expected, and is methodologically important because the tracheal system is an often-understudied character system in development. In the future, this form of time-lapse CT-scanning could allow faster and more detailed developmental studies on a wider range of taxa than is presently possible.

## 1. Introduction

Endopterygote insects—a monophyletic group which are united by complete metamorphosis with internal wing and genitalia development in larval stages—are the most successful living organisms in both species diversity and abundance [1]. One key factor in their success appears to be their life cycle, which includes complete metamorphosis, and hence differentiation between juvenile and adult forms [2]. This facilitates ontogenetic specialization including diet, reducing competition between juveniles and adults and also more effective control of development [3]. To date, the study of metamorphosis in the endopterygotes has been limited to select model organisms such as the fruitfly (*Drosophila melanogaster*; [4–6]) and blowflies (*Calliphora*; [7–9]). This is in part due to the difficult and challenging nature of traditional histological methods, which also make quantitative analysis difficult [10]. Such methods are also destructive, necessitating a single study per specimen, and requiring in ontogenetic research that multiple specimens are destructively sampled at different developmental stages. Pupae develop at different rates, and this makes it difficult to provide assurance that these isolated temporal snapshots provide an accurate picture of insect development [11,12]. A method by which the same specimen can be non-destructively investigated throughout development would overcome such limitations. High-resolution computed tomography (micro-CT, μCT) could be such a method [13–22], yet it is one which has not previously been applied in this manner to insect development; rather, the limited past studies of insect development via CT have relied on staining to gain the necessary contrast [10]. This is destructive, and thus prevents longitudinal or temporal study of a single specimen. As a chrysalis is immobile, micro-CT is an ideal technique to study metamorphosis, allowing a single specimen to be scanned throughout development. This study uses micro-CT to document the development of a Painted Lady (*Vanessa cardui* (L.)) chrysalis, revealing novel details of Lepidoptera development, and opening new avenues of research for the developmental studies of animals.

## 2. Results

**Figure 1.**
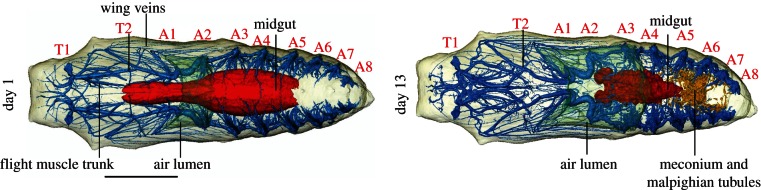
CT-based reconstructions of the chrysalis at day 1 and 13 of its development, numerous aspects of the morphology labelled. T1–T2, thoracic spiracles 1–2; A1–A8, abdominal spiracles 1–8. Scale bar, 5 mm.

**Figure 2.**
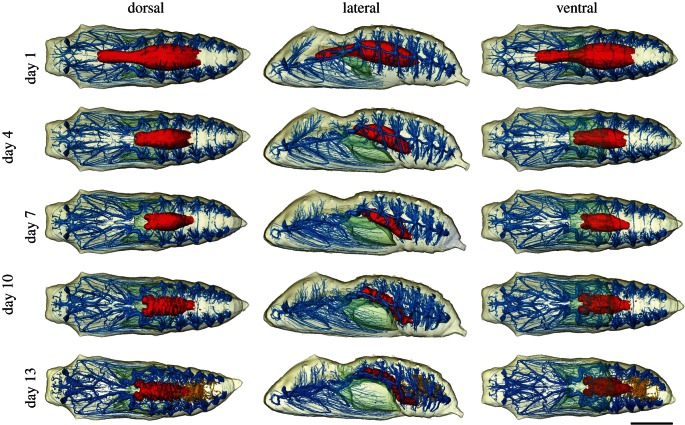
Reconstructions of the chrysalis on multiple days through development from the 1st to the 13th. Tracheal system shown in blue, midgut in red and Malpighian tubules in orange. Air lumen in transparent green, and external surface in transparent beige. Scale bar, 5 mm.

This study has demonstrated the efficacy of X-ray micro-tomography for longitudinal, *in vivo* imaging of insect metamorphosis (figure 1). It has revealed—in three dimensions at various stages in development—a number of the organ systems, principally the tracheae and portions of the gut, allowing their development to be tracked throughout development (figure 2). This was facilitated by a method aimed at minimizing radiation exposure. Combined with a naturally high radiation-tolerance in the insects (although see §3 for limitations). It is apparent that pupae can be scanned regularly throughout their development—a number hatched successfully after repeated scans (table 1). In addition to images, electronic supplementary material is available for download from the Dryad data repository [23]. Here, the models are presented as animations (see the electronic supplementary material, S1 and S2). The method facilitates a quantitative assessment of metamorphosis: the volume of the organ systems are presented in the electronic supplementary material, S3. High-resolution figure versions of the figures (see the electronic supplementary material, S4), and the Avizo surfaces of the models (see the electronic supplementary material, S5) are also available. Owing to file size limitations, no repository capable of archiving the entirety of the voxel data for these scans currently exists—in lieu, these data will be provided by either of the corresponding authors on request. The results are presented below, split into sections for each organ system.

**Table 1.** Summary of the scans performed on the chrysalis samples. X represents a scan, D indicates specimen death and H indicates specimen hatching from the chrysalis.

day	specimen									
	1	2	3	4	5	6	7	8	9	10
1	X	X	X		X					X
2										X
3										X
4	X	X			X					X
5			X							X
6				X						X
7	X	X								X
8										
9			X	X				X		
10	X	X				X		X		
11			X	X		X		X		
12							X	X		
13	X	X			H	H		X	H	
14	D	X	X	XH			XH	XH		
15		X	X							
16		XD	D							D

### 2.1. Tracheal system

Scans reveal that the majority of the adult tracheal system is well formed from the first day of pupation (figures 1 and 2). Eight abdominal spiracles and two thoracic spiracles are present. As is the case in Lepidoptera in general [24–26], the first thoracic and first abdominal spiracles have been displaced forward. As a result, the mesothoracic spiracles open on the prothorax, and the first abdominal spiracles open into the light-blue air gap at the thorax/abdomen boundary (figure 2). Internally, radiating from each abdominal spiracle is a dendritic network of tracheae. Thoracic tracheae are longer and more continuous. Those in the limbs, wings and even antennae and proboscis are already present. The tracheal system displays no major changes in morphology throughout development. Cephalic structures do become better formed (most notably between days 1 and 4), and the thoracic tracheal systems become gradually more complex: the primary tracheal trunks increase in size, and a number of new branches develop. These are largely small, morphologically complex, anastomosing tracheae, increasing tracheal presence towards the median axis of the chrysalis. In particular, the flight muscle trunk and associated branches increase in volume and—in the latter—number. Interestingly, the flight muscle trunk appears to originate from the first thoracic spiracle contrary to Wasserthal [26, fig. 7.5] and Srivastava [24], where it is reported and shown to originate from the second thoracic spiracle. Other than this, the development and outline of the tracheal system corresponds to Srivastava [24] and Wasserthal [26], but lacks large air sacs dorsally in the abdomen, thoracic base and head. These develop very late and only inflate just before or after the adult emerges from the pupa, however [26]—i.e. after our last scan. Electronic supplementary material, S3 includes the change in tracheal volume as a percentage of chrysalis volume, demonstrating a gradual increase in throughout development.

### 2.2. Air lumen

**Figure 3.**
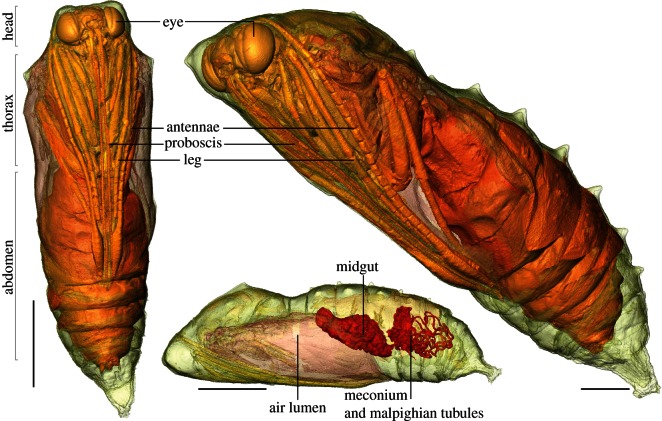
The pharate adult at 16 days development, showing aspects of the internal anatomy (air lumen and gut structures), and the external anatomy such as limbs, mouthparts and the cuticle. Scale bars, 5 mm.

Within the chrysalis a U-shaped ventral air gap is present at the boundary between the thorax and abdomen from day 1 (figures 1 and 2). This grows at a steady rate throughout development. This is the sole large airspace within the cuticle until the 10th day of development. By the 13th day of development, a number of smaller dorsal air sacs become visible around the thorax and abdomen (figure 2), which, by hatching, have expanded and combined into the air space around the pharate adult form (i.e. the adult prior to emergence from the pupa; figure 3). At day 16, an inflated median abdominal air sac level with spiracles six and seven appears. This is particularly clear in the electronic supplementary material, video S2.

### 2.3. Gut

The scans clearly show the transformation from an elongate, sausage-shaped larval midgut to the much shorter adult midgut. The full larval midgut is only present in scans from day 1; on day 4, the narrower anterior midgut has disappeared, but the remaining posterior remains similar in shape and position. By day 7, the midgut has moved backwards to its final position and is more complex in form with two anterior bilaterally symmetrical outgrowths and a posterior convolute surface (figure 2). As demonstrated by the volume measurements in the electronic supplementary material, S3, there is initially a gradual decline in the volume of the gut visible in scans. This is true for the first week of development, as the midgut contracts. From day 7 (and discounting the anomalous result on day 14), the volume of the segmented gut increases again, in part as it lengthens, and then by day 13 as the meconium and Malpighian tubules can be differentiated, and are included in the gut volume measurement. The backwards displacement and strong reduction in size of the adult midgut compared with the larva are both apomorphies for higher Lepidoptera [27]. At day 10, the midgut has a convolute surface; the two anterior lobes are followed by around 10 roughly bilaterally symmetrical wrinkles. By this point, the gut also possesses a terminal narrowing, and has repositioned from sub-horizontal to a 45° angle to accommodate the thorax–abdomen boundary (figure 2). In the next 3 days, the midgut is significantly more wrinkled, and the termination is even narrower. The two anterior lobes, the strongly wrinkled outer surface and the terminal narrowing corresponds exactly to earlier observations on the congeneric *Vanessa indica* based on light microscopy by Homma ([28, fig. 51]). Immediately, posterior and dorsal to the termination is the meconium, which can now be identified in scans (figures 2 and 3), and the Malpighian tubules which reforms in the later stages of the pupa development [29]. Between day 13 and 16, the cuticle-lined gut shortens markedly, becoming more bulbous and thicker.

### 2.4. Pharate adult body wall cuticle

Scans on and before day 13 do not reveal any of the cuticle, suggesting sclerotization is poor prior to this stage of the development. The cuticle of the insect only becomes apparent in scans on day 16, when the fully developed pharate adult form—including appendages, eyes, mouthparts and abdomen—is present (figure 3).

## 3. Discussion

### 3.1. Tracheal system

According to Wasserthal [26], the pupal tracheal system is reduced (i.e. less voluminous) compared with that of both caterpillars and adults. Our results suggest a slightly more complex picture. It is clear that there is considerable difference between the young and the mature pupa. The volume of the tracheal system increases dramatically during pupal development (see the electronic supplementary material, S3). However, the ground plan of the adult tracheal system is present in the early stages of pupal development as demonstrated by the presence of tracheae in the wings, legs, antennae and proboscis in day 1. If air sacs similar to those reported in adults [24,26] are added to the volume reported here, tracheal system in adults is indeed much greater in volume than that of the pupa.

Micro-CT allows the tracheal system to be reconstructed in considerable detail, and the quantitative measurement of tracheal system growth during pupal development has, to our knowledge, not been documented before. This may well be the most significant result of this paper, because tracheal systems are one of the most understudied character systems in insects in general [24,25] both in comparative and developmental work. Our results demonstrate that micro-CT is a very powerful tool for precisely reconstructing even very fine branches of insect tracheae. It thus provides an excellent opportunity for relatively easy comparison between life stages for developmental studies and evolutionary comparisons between various taxonomic groups.

### 3.2. Midgut

The development of the midgut follows the patterns reported elsewhere [27]. Very early in the development, we still see the large larval midgut, but the size is quickly reduced, and the midgut moved backwards to reach its final position, size and general morphology by day 7. Our method is also effective for identifying less conspicuous structural changes such as the development and presence of the lateral anterior lobes characteristic of many Nymphalidae ([28, fig. 35–53]). The reason why only midgut development can be observed is likely that this section is comparatively thick-walled with large cells active in digestion compared with the thin-walled, cuticle-lined fore- and hindguts [27]. These results make it clear that the majority of morphological change happens before day 7—with more frequent scans, micro-CT could serve as a powerful tool for pinpointing exactly when in the development these major changes occur.

### 3.3. Limitations

There are a number of limitations when applying micro-CT to insects. A number of tissues—for example, the muscles and central nervous system—are not resolved in the current scans due to lack of contrast. Thus, to use micro-CT to investigate the development of these important organs and systems, staining is required [10], which precludes temporal studies of a single individual. Phase-contrast approaches—while currently requiring a synchrotron—could help in this regard [15,30]. In the meantime, for these parts of the anatomy, micro-CT can be used to calibrate developmental stages in comprehensive studies. A lack of contrast, even in stained preparations, as reported by Richards *et al*. [10], emphasizes the importance of continued teaching of and research into insect comparative morphology and histology: such experience is the only reliable way to differentiate structures in micro-CT scans of insects.

Ionizing radiation causes tissue damage [31] and thus risks altering the development of the specimen when repeatedly scanned. To counter this, steps were taken to minimize exposure in this study (§5), which were aided by the fact that insects are very radiation-tolerant [32], even as pharate adults [33]. That a number of the scanned insects hatched after the standard pupation period suggests radiation effects were moderate—although it appears that those scanned prior to the 6th day after pupation were more likely to show detrimental effects (this could be due to radiation damage, or internal heating during scans).

### 3.4. Future directions

In the future, CT-based studies of insect development will include the widespread application of the technique to different groups. This could be especially valuable for taxa more distantly related to model organisms, whose development is likely to differ from, and be less well studied, than model endopterygotes [29]. Furthermore, the technique will allow easier study of, for example, development in mutants of model organisms such as *Drosophila*, and the impact these mutations have on development. Other possibilities could include the impact of environment and ecotoxicological effects on wild insects. Finally, the power of three-dimensional reconstruction as a tool for science education and outreach is becoming increasingly recognized [34], at a time when there is an increasing creationist threat [35]. This often focuses on complex systems which are hard to analyse, calling on—for example—flawed arguments such as irreducible complexity [36]. Micro-CT of these very processes could provide a widely available and attractive educational tool to help counter creationist disinformation.

## 4. Conclusions

The application of micro-CT is a novel means to study and quantify insect metamorphosis. It allows the longitudinal, *in vivo* study of a single individual, and facilitates comparison between taxa for systematic research more quickly, with less effort, than using traditional techniques (but still calls for the training of traditional histologists). For a significant portion of the anatomy, it does not require staining and is non-destructive. Furthermore, the technique is increasingly widely available and affordable. Its widespread application would provide a new window for studying insect development, allowing studies in a range of taxa, and breaking the current reliance on a limited number of model organisms.

## 5. Method and materials

### 5.1. Materials

Nine *Vanessa cardui* (L.) (Lepidoptera: Nymphalidae, Painted Lady) pupae were scanned: two at 3 day intervals throughout development; one daily for the first 7 days; one daily for the last 6 days of development, and five a limited number of times at various points during development to act as references (table 1). One further specimen was not scanned. One specimen was successfully scanned as an adult, while attempts to scan a live caterpillar shortly before pupation were unsuccessful due to specimen movement. The results presented herein are from specimen 2 which presented the most complete picture of development, and were verified against scans the other specimens.

### 5.2. Micro-computed tomography

Between scans specimens were suspended from their cremaster. They were placed vertically on a polymer tube support during scanning. CT was conducted on a Nikon Metris 225/320 kV X-ray CT housed in a customized bay. The detector on the chosen system (2000 × 2000 Perkin Elmer 1621-16-bit amorphous silicon flat-panel) allows finer differences in contrast to be observed than the standard, facilitating shorter scan times and minimizing specimens’ radiation exposure. The system also permits target materials to be changed for optimization of the X-ray density at any given energy range. These scans were conducted at 45 kV with a molybdenum target, producing the highest X-ray density at this voltage and improving signal to noise ratio. Scans were conducted at 450–500 μA, with 500 ms exposure for 1901 projections, and a gain of 32. A source to object distance of 69 mm and source to detector distance of 1007 mm was employed.

### 5.3. Visualization

The Nikon Metris CT-Pro software was used to reconstruct datasets with a voxel size of 13.1–13.8 μm based on attenuation contrast. After reconstruction, the data were loaded into Avizo standard 7.0 (Visualization Sciences Group (VSG), Bordeaux, France) for examination of the virtual slices and three-dimensional volume renderings. This software was used to segment the midgut, tracheal system, meconium and Malpighian tubules, air lumen and external morphology based upon their grey scale values, primarily using a thresholding technique. In regions where the standard thresholding technique did not work due to poor contrast, features were selected using the magic wand tools in conjunction with a redefined grey scale range within the local region. These volumes were then used in the Avizo standard analysis software (VSG) in order to determine their respective volumes.
